# The Macklin Effect: An Underestimated Cause of Pneumomediastinum

**DOI:** 10.7759/cureus.69328

**Published:** 2024-09-13

**Authors:** Bryan A Morales Eslava, Julio E Suástegui Mares, Gonzalo A Chuc Baeza, Ana Sánchez Arzate

**Affiliations:** 1 General Surgery, Universidad Juárez Autónoma de Tabasco, Villahermosa, MEX; 2 General Surgery, Universidad Popular Autónoma del Estado de Puebla, Puebla de Zaragoza, MEX

**Keywords:** blunt trauma, blunt chest trauma, spontaneous pneumomediastinum (spm), macklin effect, pneumomediastinum (pm), macklin phenomenon

## Abstract

The Macklin effect is a rare but potentially serious complication of pneumomediastinum, caused by the dissemination of air from the lungs into the subcutaneous tissue and mediastinum after severe chest trauma or invasive manipulation. Early recognition is crucial for proper management of the patient.

A 33-year-old male skidded while riding a motorcycle, lost control of the vehicle, and crashed into a utility pole with a thoracic contusion. He was admitted to the hospital; a computed tomography (CT) of the chest and abdomen was requested, which ruled out the presence of fractures and showed air in the mediastinum and subcutaneous cellular tissue, with features suggestive of the Macklin phenomenon. After 72 hours of inpatient monitoring, the patient was discharged to the general surgery outpatient clinic.

The Macklin phenomenon occurs as a result of airway rupture due to negative pressure caused by trauma or invasive mechanical ventilation. Risk factors include a higher prevalence in young males, a slender stature and above-average height, and an age range of 12 to 35 years. Early detection of the Macklin phenomenon is crucial to recognize and prevent further complications.

This case demonstrated the importance of considering the Macklin effect as a cause of pneumomediastinum in patients with severe chest trauma. Diagnostic imaging plays a key role in confirming the diagnosis and planning treatment.

## Introduction

Pneumomediastinum is a finding with multifactorial etiology, defined as the presence of free air or gas in the mediastinum [1]. The Macklin effect is a form of pneumomediastinum caused by the dissemination of air from the lungs into the subcutaneous tissue and mediastinum, which usually occurs after severe chest trauma or invasive manipulation [2, 3].

In 1944, Macklin defined the pathophysiologic process of this effect, identifying increased alveolar pressure as the basic mechanism of this event, which causes air rupture and escape through the pulmonary interstitium, spreading through the bronchovascular spaces to the pulmonary hilum and finally to the mediastinum [2].

Although diagnosis can be difficult, early recognition is crucial for the proper management of the patient [4]. The present case describes a 33-year-old male patient who developed the Macklin phenomenon after a motorcycle accident with chest trauma. The aim is to demonstrate the clinical and radiologic features of the Macklin phenomenon, a rare but potentially serious complication.

## Case presentation

**Figure 1:**
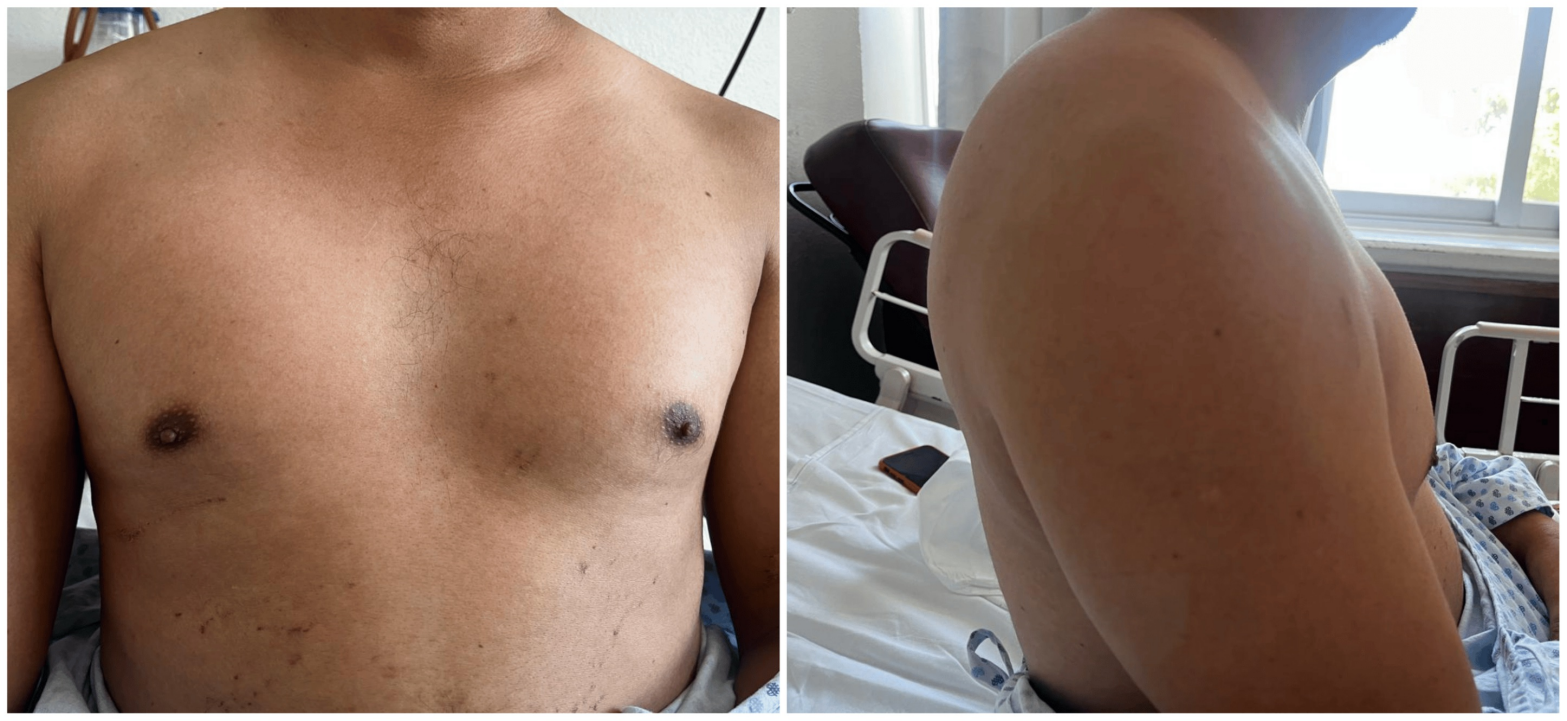
Anterior and lateral view during physical examination of the patient

A 33-year-old man skidded his motorcycle, crashed into a utility pole, and suffered chest trauma. He was transferred to our hospital. On admission, he had a heart rate of 80 beats per minute, blood pressure of 110/80 mmHg, respiratory rate of 24 breaths per minute, and pulse oximetry saturation of 96% without supplemental oxygen. He was conscious, reported chest pain, and denied dyspnea. On examination, he had a symmetrical chest and adequate respiratory movements. No fractures or hematomas were noted. Palpation revealed subcutaneous emphysema in the sternal and parasternal region on both sides with mild pain on manipulation. Auscultation revealed diminished breath sounds on both sides and bilateral basal rales. The heart sounds were normal (Figure 1).

**Figure 2:**
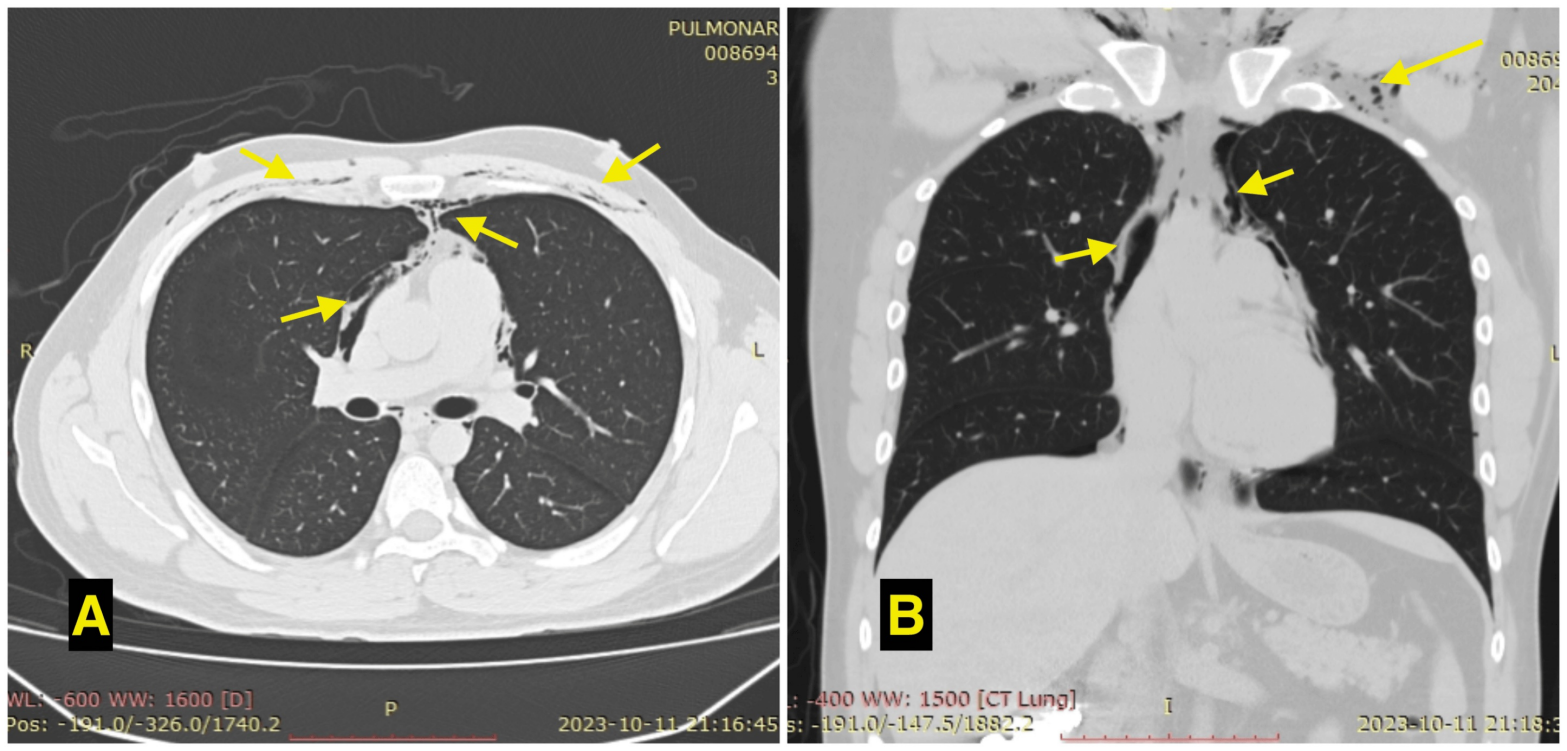
A: CT scan in axial view; the arrows point to pneumomediastinum and subcutaneous emphysema. B: CT scan in coronal view; the arrows point to pneumomediastinum and subcutaneous emphysema.

A computed tomography (CT) of the chest and abdomen was requested, which ruled out the presence of fractures and showed air in the mediastinum and subcutaneous cellular tissue, with features suggestive of the Macklin phenomenon (Figure 2). As a complementary study, an esophagogram was requested, which showed no esophageal injury. Conservative treatment with bed rest, analgesics, and breathing exercises using an incentive spirometer was indicated. Clinical evolution was adequate, and subcutaneous emphysema decreased. After 72 hours of inpatient monitoring, it was decided to discharge him to the general surgery outpatient clinic.

**Figure 3:**
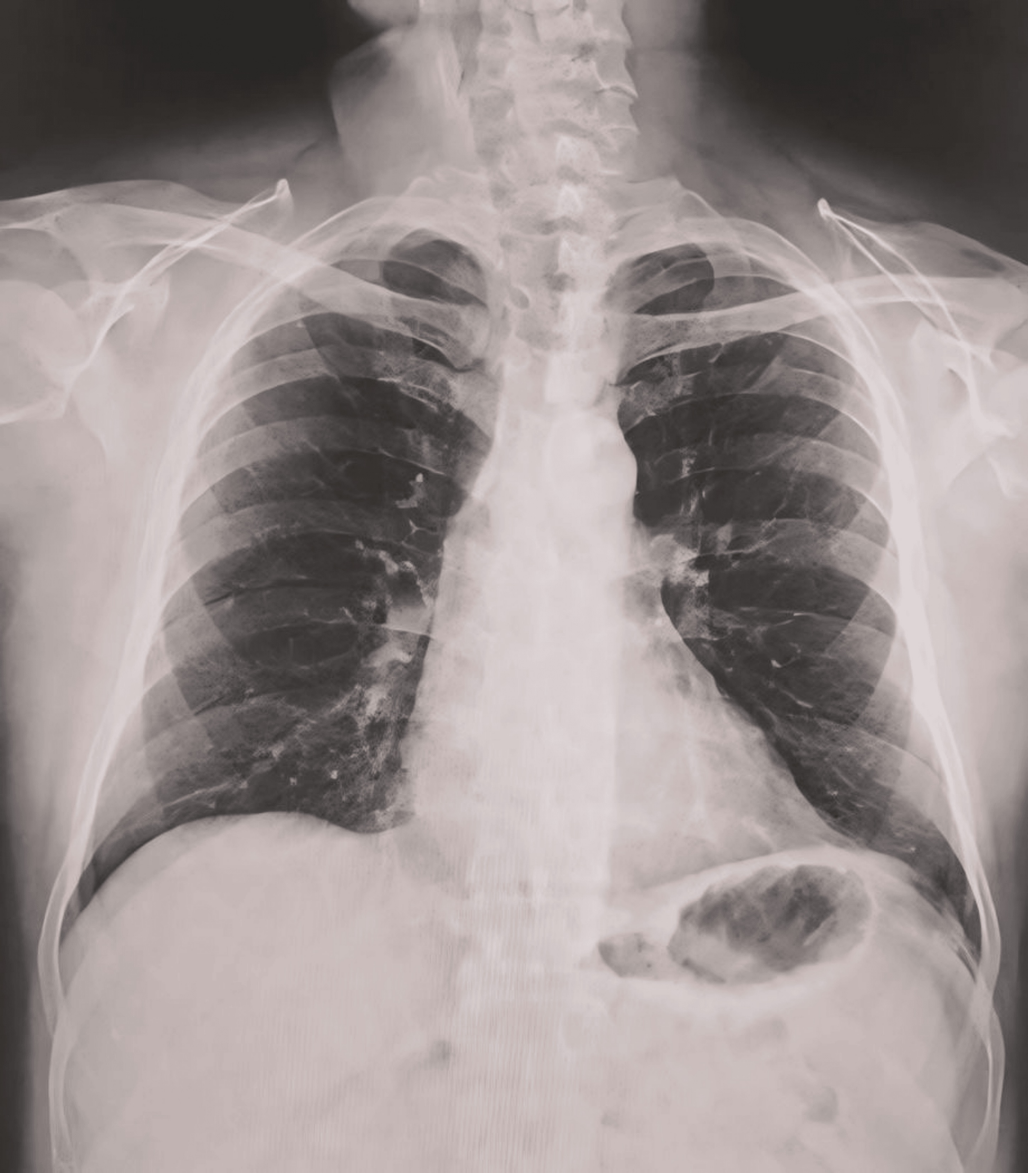
The chest X-ray shows no evidence of pneumothorax or pneumomediastinum.

He was re-examined four days after discharge from the hospital and was found to be asymptomatic. On physical examination, vital signs were in the normal range, chest was symmetrical, respiratory movements were adequate, auscultation revealed normal vesicular breath, oxygen saturation was 99% without supplemental oxygen, and chest X-ray showed no evidence of pneumothorax or pneumomediastinum (Figure 3).

## Discussion

The Macklin effect occurs as a result of airway rupture due to negative pressure caused by trauma or invasive mechanical ventilation [2, 3]. Some risk factors have been identified, such as a higher prevalence in men, an age range of 27 to 55 years, and a high association with traffic accidents [3]. Our patient was no exception to these statistics, as he was a 33-year-old male with a history of chest trauma that caused an alveolar rupture and allowed the spread of air through the subcutaneous tissue into the mediastinum.

Early detection of the Macklin effect is crucial to recognize and prevent further complications such as extensive subcutaneous emphysema, pneumothorax, tamponade, and pneumopericardium, which usually require other interventions including skin incisions, chest tube drainage, video-assisted thoracoscopic surgery (VATS), or even thoracotomy [3, 4]. As part of the diagnostic approach, a CT scan plays an important role as it detects air collections that spread along the bronchovascular sheaths to the hilum and into the mediastinum [5]. In addition, a CT scan can help to differentiate other etiologies of pneumomediastinum and rule out associated injuries such as fractures, hemothorax, or pneumothorax [1, 5]. In the present case, alveolar air was visualized in the mediastinum and perivascularly, confirming the involvement of the Macklin phenomenon.

Treatment in stable patients is conservative and includes rest, analgesics, and avoidance of Valsalva maneuvers. However, depending on the patient's concomitant injuries, further therapeutic interventions such as chest tube placement may be necessary [4]. To avoid overtreatment, Okada et al. proposed a checklist with five questions consisting of the following points: Fever >38°C, oxygen saturation of less than 96%, progressive symptoms, vomiting, and presence of anxiety [6]. As this case was a clinically stable patient who did not meet any of the above criteria, conservative treatment was chosen and an outpatient approach was preferred.

## Conclusions

This case demonstrates the importance of considering the Macklin effect as a cause of pneumomediastinum in patients with severe chest trauma. Clinical presentation may vary, but diagnostic imaging plays a key role in confirming the diagnosis and planning treatment.

This rare phenomenon should be recognized early because, despite its usually benign course, if properly identified, unnecessary invasive diagnostic or therapeutic procedures can be avoided, improving outcomes and reducing associated morbidity.
